# Hesperidin protects against aluminum-induced renal injury in rats via modulating MMP-9 and apoptosis: biochemical, histological, and ultrastructural study

**DOI:** 10.1007/s11356-022-24800-0

**Published:** 2022-12-22

**Authors:** Nancy Husseiny Hassan, Doaa Mohammed Yousef, Amira Ebrahim Alsemeh

**Affiliations:** Human Anatomy and Embryology Department, Faculty of Medicine, Zagazig University, Zagazig, 44519 Egypt

**Keywords:** ALCL3, Hesperidin, Kidney, MMP-9, FAS, EGTI scoring system

## Abstract

Aluminum, one of the most abundant metallic elements, is known to be toxic to multiple organs including the kidneys. This study aimed to investigate the pleiotropic nephroprotective effects of Hesperidin in aluminum chloride (ALCL3)-induced renal injury, highlighting the potential molecular mechanisms underlying. Twenty-four male albino rats were divided into four groups: control, Hesperidin (80 mg/kg BW, orally), ALCL3 (10 mg/kg BW, IP), and ALCL3 + Hesperidin groups. By the end of the study, blood samples were collected, and tissue samples were harvested at sacrifice. ALCL3 rats showed dramatically declined renal function, enhanced intrarenal oxidative stress, inflammation, apoptosis, and extravagant renal histopathological damage with interstitial fibrosis as shown by a higher Endothelial, Glomerular, Tubular, and Interstitial (EGTI) score. Hesperidin significantly reversed all the aforementioned detrimental effects in ALCL3-treated rats. The study verified the nephroprotective effects of Hesperidin on ALCL3-induced renal damage and confirmed the critical role of extracellular matrix (ECM) remodeling and apoptosis inhibition.

**Graphical Abstract:**

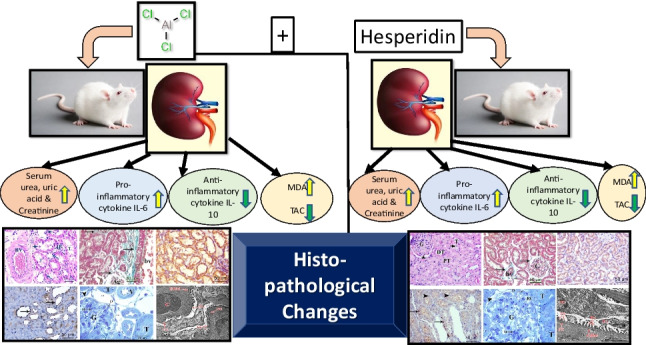

## Introduction

Aluminum is one of the most plentiful metallic components on the planet; it establishes around 8% of the complete mineral substance of the world outside. Recently, increased attention to the biotoxicity of aluminum is considered due to its availability (Al Eisa and Al Nahari 2016). Aluminum is used as food additive. Moreover, aluminum pots represent around 20% of the relative multitude of pots on the planet. Aluminum is additionally present in water suitable for human use with a convergence of 0.2 mg/L, water-filtering specialists, jars and containers, foil paper made from aluminum, and beauty care products (Fatima et al. 2016). Thusly, human beings are highly susceptible to aluminum toxicity as it may accumulate in the kidney causing nephrotoxicity due to its high availability (Fatima et al. 2016). Additionally, aluminum can liberate receptive oxygen species (ROS) and apoptosis by animating the favorable to oxidant effects of iron and copper, resulting in mitochondrial dysfunction and subsequent oxidative deterioration of macromolecules and releasing of cytochrome C from the mitochondria (Al-Olayan et al. 2015; Zahedi-Amiri et al. 2019).

Renal function contributes to the elimination of aluminum, such as aluminum chloride (ALCL3) via glomerular filtration, reabsorption of filtrated ALCL3 in tubules, secretion, and excretion in distal tubules (al Kahtani 2010). The extreme exposure of aluminum due to dissimilar human daily lifestyles raised the threat of renal aluminum withholding due to the accumulation of aluminum to renal tubules resulting in renal dysfunction (Al Dera 2016; Hasona and Ahmed 2017).

According to this background, the kidney is a vigorous tissue that is more liable to toxic insults of ALCL3 which impairs the pro-oxidant/antioxidant stability, which motivates the pro-oxidant effects of iron or copper (Zahedi-Amiri et al. 2019). This augments the lipid peroxidation (LPO) process and decreases the actions of the antioxidants, which in turn overgenerates oxidative stress, leading to renal toxicity. Therefore, antioxidant compounds that can mitigate the oxidative insult may be a nominee to relieve ALCL3 toxicity (Hasona and Ahmed 2017).

Hesperidin—a flavanone glycoside—is a cheap rich byproduct of citrus cultivation (Rushdy et al. 2012). Hesperidin displayed an obvious superoxide radical scavenging action (Cho 2006; Kumar et al. 2011). Hesperidin is a potential antioxidant mediator against free radicals and justifies clinical trials (Wilmsen et al. 2005; Balakrishnan and Menon 2007). Mishra (2013) reported Hesperidin’s effects on antioxidative properties, examined with a free-radical scavenging system, including reducing power, chelating activity on Fe^2+^ free radical scavenging, hydrogen peroxide scavenging, and hydroxyl radical scavenging activities, in an attempt to understand its mechanism of action which may pave the way for possible therapeutic applications (Pradeep et al. 2008; Kamaraj et al. 2009).

Also, a high expression of MMP-9 can make an epithelial-mesenchymal transformation (EMT) in tubular cells, which could be an additional mechanism for the induction of renal fibrosis (Yang et al. 2002a^a^; Tan et al. 2013).

According to the previously stated properties of Hesperidin, we designed this work to clarify Hesperidin’s potential effect in modifying the probable biochemical, histological, and immunohistochemical renal deterioration caused by ALCL3.

## Material and methods

### Test chemicals

- Aluminum chloride (ALCL3), Hesperidin, and additional routine chemicals (analytical grade) were purchased from Sigma-Aldrich® Chemical Company (St. Louis, MO, USA).

### Experimental animals

Twenty-four male Wistar rats (6 weeks old, with weight of 180–200 g) were accustomed for one week before ALCL3 administration and afterward randomly distributed into four groups (*n* = 6). The rats were housed in the Animal House, Faculty of Medicine Zagazig University, with access to food and water ad libitum. The housing conditions were maintained at a constant temperature (24 ± 1 °C), relative humidity (55 ± 5%), ventilation frequency (18 times/h), and a 12 h light/dark cycle. The rats were housed in plastic cages (four rats per cage) with soft chip bedding. The size of the cage was 47 × 30 × 15 cm, which was large enough for the growth of four rats. Throughout the experiment, the wood chips were renewed every 3 days. The health status of the rats was monitored daily. All measures to minimize pain or discomfort were taken by the investigators. The Institutional Animal Care and Use Committee of Zagazig University accepted the rat experiments (approval no. ZU-IACUC/3/F/79/2020). All maneuvers followed the guidelines recognized in the Guidelines for the Care and Use of Laboratory Animals.

### Experimental strategy

Twenty-four rats were allocated to the following groups:

Control group: contained 6 animals injected with sterile physiological saline intraperitoneally for 5 weeks.

Hesperidin group: the 6 rats were orally treated with Hesperidin only (80 mg/kg body weight) dissolved in dimethyl sulfoxide (DMSO), by gavage for 5 weeks (Pari et al. 2015).

Aluminum chloride (ALCL3) group: which included 6 rats that were given intraperitoneally 10 mg/kg body weight of ALCL3 diluted in normal saline every day for 5 weeks, 1 g dissolved in 500 mL distilled water. So, each mL of distilled water contains 2 mg of ALCL3 (Mostafa et al. 2015).

Aluminum chloride (ALCL3) + Hesperidin group: included 6 rats; each rat was injected intraperitoneally with 10 mg/kg BW of ALCL3 in concomitant with the oral intake of Hesperidin 80 mg/kg once daily by gavage for 5 weeks.

### Tissue sampling

By the end of the experiment, animals were exposed to fasting over the night. In the morning, they were anesthetized by a single intraperitoneal (I.P.) injection of thiopental (75 mg/kg/BW) (Tardif et al. 2013). Blood samples were directly taken from the retro-orbital venous sinuses of the rats in each group (Parasuraman et al. 2015). The samples were kept to coagulate at room temperature and then centrifuged at 3000 R.P.M. for 10 min. The serum was collected and frozen at − 20 °C for the urea, creatinine, and uric acid biochemical analysis in serum. After that, laparotomy was done, and the right and left kidneys were sensibly dissected and rapidly isolated from each group. The right kidneys of the animals in each group were used for histopathological examination, so they were prepared in 10% neutral buffered formalin solution for 2 h to be hardened. The left renal specimens of each group were divided into two portions; one portion was preserved at − 80 °C for further homogenization. They were homogenized in 50 mM Tris–HCl pH 7.4 and 300 mM sucrose, making up 10% (w/v) homogenate with a tissue homogenizer (Heidolph Instruments, Donau, Germany). The obtained homogenate was centrifuged at 4000 R.P.M. for 15 min. at 4 °C, and the supernatant was utilized for the malondialdehyde (MDA) estimation, the total antioxidant capacity (TAC), and the inflammation marker (Interleukin-6 and 10). The other portion of the left kidney specimens was processed for electron microscopic examination; renal cortex samples (1 mm thickness) were preserved in a mix of 2.5% glutaraldehyde and 2.5% paraformaldehyde.

### Biochemical analysis

#### Measurement of serum urea, creatinine, and uric acid

Sera were collected and kept in aliquots at − 20 °C until they were utilized to estimate serum urea, uric acid, and creatinine levels. Their readings were estimated with commercially accessible kits as stated by the manufacturer’s directions (SPECTRUM Diagnostic kits were acquired by the Egyptian Company for Biotechnology (S.A.E.), Obour city industrial area, block 20,008-piece 19 A. Cairo, Egypt).

#### Assessment of the proinflammatory cytokines (IL-6) and the anti-inflammatory cytokines (IL-10) in renal tissue homogenates

Proinflammatory cytokine IL-6 and anti-inflammatory cytokine IL-10 concentration was assessed in renal samples frozen at − 80 C. IL-6 (SEA079Ra) was assessed with RAT Platinum Enzyme-Linked Immunosorbent Assay Kits (Wuhan USCN Business Co. Ltd. Houston, TX) according to the manufacturer’s directions with an absorbance reader (BioTek ELx800, BioTek, Winooski, VT) at 450 nm. These results were considered by the four-parameter curve method and stated as picograms (pg)/gm tissue.

#### Assessment of tissue malondialdehyde (MDA) and total antioxidant capacity (TAC) in renal tissue homogenates

The degree of lipid peroxidation was checked by estimating the degree of MDA utilizing commercial kits as defined by Ohkawa et al. (1979). The method depends on the interaction of thiobarbituric acid with MDA in acidic medium at a temperature of 95 °C to form thiobarbituric acid reactive product, and the absorbance of the resultant pink product was measured at 534 nm.

The TAC was estimated using commercial kits bought from Bio-diagnostics dependent on the technique depicted by Koracevic et al. (2001). The antioxidative capacity was performed by the antioxidants’ reaction in the specimen with a characterized measure of an exogenously given hydrogen peroxide (H_2_O_2_). The antioxidants in the sample eliminated a certain amount of the provided hydrogen peroxide. The residual H_2_O_2_ was determined colorimetrically by an enzymatic reaction which involves the conversion of 3,5-dichloro2-hydroxy benzene sulfonate to a colored product. The color was measured spectrophotometrically at 505 nm. Protein content in tissue homogenate was measured according to the method of (Lowry et al. 1951).

### Real-time polymerase chain reaction (RT–PCR) analysis for MMP-9 gene expression in renal tissues

Real-time PCR RNA was used to detect the mRNA expression of MMP-9. Total RNAs were isolated using an RNA commercial kit (Intron, Sungnam, Korea) according to the manufacturer’s instructions. The extracted RNA was reverse transcribed by QuantiTect RT–PCR kit (Qiagen; catalog no. 204243) to form cDNA as recommended by the manufacturer. The quantity and quality of the RNA were confirmed by measuring the A260/A280 percentage. The meditation and purity of RNA were assessed using a spectrophotometer (UNICO, UV2000, China). Glyceraldehyde-3- phosphate dehydrogenase (GAPDH) was used as a reference gene for standardizing the expression data as presented in Table 1 (Zawada et al. 2015).

**Table 1:** Primer sequences

Gene	Primer 3′ → 5′	Reverse primer 3 → 5’	Pb	Accession no
MMP-9	CAAACCCTGCGTATTTCC	AGAGTACTGCTTGCCCAGGA	223	NM_031055.2
Gapdh	CTCATGACCACAGTCCATGC	TTCATCGGGATGACCTT	152	NM_001394060.2

### Histological assessment by light microscopic examination

#### General histopathological characteristics by hematoxylin and eosin staining

All procedures were performed in the Department of Pathology, Faculty of Medicine, Zagazig University. As per usual methodology (Suvarna et al. 2018), the right renal samples were secured in 10% neutral buffered formalin and inserted in paraffin. Sections of 5 μm thickness were mounted on glass slides, deparaffinized in xylene, and stained utilizing hematoxylin and eosin stain (H&E).

#### Histopathological scoring

Renal sections were examined and scored to assess the degree of injury. A skilled histopathologist, who was blinded to the group allocation, assessed histological damage and measured it with the Endothelial, Glomerular, Tubular, and Interstitial (EGTI) scoring system developed precisely for animal studies on kidney tissue in the context of injury (Table 2). The scoring of the renal cortex was made in all studied groups of the renal cortex (Khalid et al. 2016).

**Table 2:**
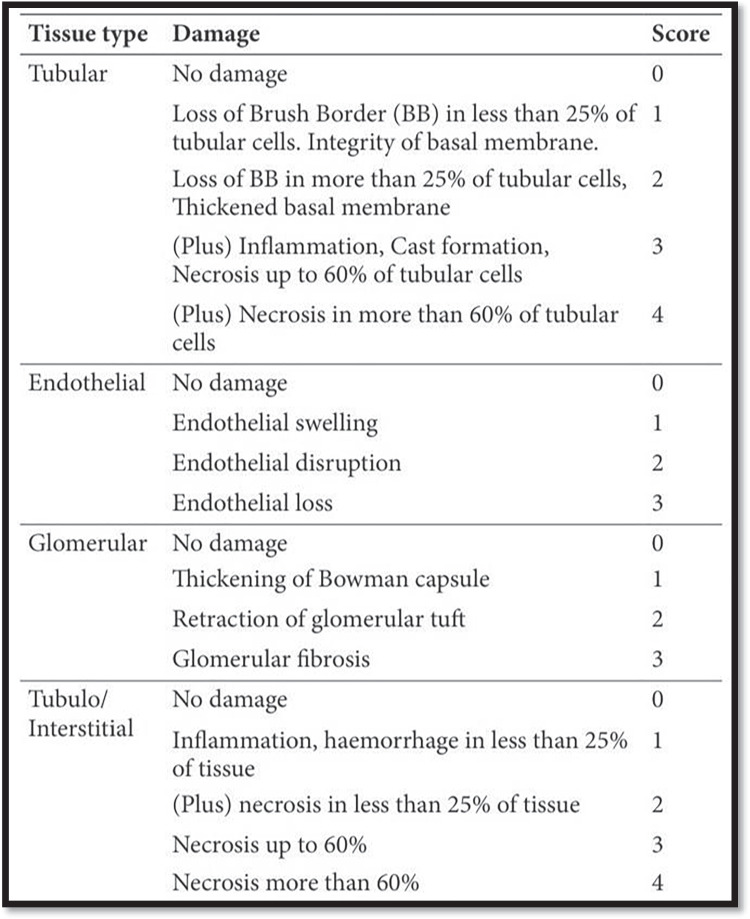
The EGTI histology scoring system (Khalid et al. 2016)

#### Histopathological evaluation of renal fibrosis

We used the paraffin blocks of formalin-fixed renal specimens in Masson’s trichrome to stain the collagen fibers and assess the area percent of fibrosis. The areas of fibrosis appeared in green while the parenchyma appeared in red color.

#### Immunohistochemistry (IHC) for assessment of matrix metalloproteinase-9 (MMP-9), (FS7-associated cell surface antigen) FAS protein, caspase-3, BAX, and BCL2 expression

We utilized the paraffin specimens of formalin-fixed renal samples for immunohistochemical examination. Paraffin sections of 4 μm thickness were prepared and mounted on positively charged slides, deparaffinized in xylene, hydrated in descendant ratings of ethanol, and treated with 3% hydrogen peroxide in methanol to block the endogenous peroxidase action. Antigens were retrieved using 0.01 mol/L citrate-buffered saline (pH 6.0), and endogenous peroxidase activity was quenched using 0.3% (v/v) H_2_O_2_ in phosphate-buffered saline. Then, the non-specific binding of immunological reagents was blocked by incubating the samples with normal goat serum 10% (v/v) for one hour. A goat anti-mouse MMP-9 antibody (Santa Cruz Biotechnology, Santa Cruz, CA, USA) diluted 1:100 was applied to ultrathin sections overnight at 4 °C, followed by a second layer of biotin-conjugated, affinity purified rabbit anti-goat immunoglobulin G (Santa Cruz) diluted 1:100 with phosphate-buffered saline and 0.1% bovine serum albumin for 15 min at room temperature, and finally by streptavidin-conjugated horseradish peroxidase, rabbit monoclonal FAS antibody (ab-15285 Abcam, Cambridge, UK) with a dilution of 1:100 in PBS for 1 h at room temperature and anti-BAX, rat monoclonal antibody (1:50, no. 13401A, clone G206-1276, immunoglobulin (Ig) M, 0.5 mg/mL, PharMingen, San Diego, CA). Additionally, anti-active caspase-3 antibody incubation (Abcam, Cambridge, MA, USA) was used. Anti-active caspase-3 antibody was diluted to 1:300. Moreover, monoclonal mouse using a Bio-Rad GS-690 densitometer and Molecular anti-rat Bcl-2 (to confirm Bcl-2 staining; Santa Cruz BioAnalyst version 4 analysis software) (Bio-Rad Laboratories chemicals) were also used. Polyclonal mega using the same analysis package and confirmed rabbit anti-rat Bcl-2 (1:400; PharMingen, San Diego, CA, by visual comparison to the ribosomal RNA subunits, USA) was diluted at 1:400. The primary antibodies were detected with a biotin-streptavidin detection system with 3,3′-Diaminobenzidine (DAB) (Sigma Aldrich, USA) as a chromogen, and then, counter-staining with Mayer’s hematoxylin was performed. Negative control segments were prepared with similar rules with replacement of the definite primary antibodies with normal rabbit immunoglobulin G (IgG) (Suvarna et al. 2018).

### Histological assessment by transmission electron microscopic (TEM) examination

The renal cortex samples (1 mm thickness) were preserved in a mix of 2.5% glutaraldehyde and 2.5% paraformaldehyde, put in phosphate buffer for 24 h, post-fixed in 1% osmium tetra-oxide, dehydrated, and fixed in resin. The samples were cut and sectioned into the semi-thin and ultrathin sections. Semi-thin Sects. (1 μm) were stained with toluidine blue and observed using a light microscope. Then, the thin slices were cut on an ultra-microtome via a diamond knife to yield the ultrathin sections of 60–90 nm thick, and they were moved to copper grids for staining with lead citrate and uranyl acetate. The sections were studied by a transmission electron microscope in Electron Microscopic Examination Unit in Mansoura University using a Zeiss EM 100 S transmission electron microscope at 60 kV to perceive the ultrastructural changes.

### Histomorphometric analysis

Morphometric examination was done on every rat in each group. After immunostaining with the anti-MMP-9, anti-FAS, anti-caspase-3, anti-BAX, and anti-BCL2 antibodies, perceptive fields across the pictures caught by the light microscope at 400 × amplification were chosen to quantify the percentage area of FAS, caspase-3, BAX, and BCL2-positive response and the optical density (pixel) of MMP-9 protein in the renal cortex from 6 rats/group. Furthermore, the percentage area of Masson’s trichrome-positive region was likewise determined in the caught discerning fields from 6 rats/group, and the mean/median values were accounted for ImageJ analysis software (Fiji ImageJ; 1.51 n, NIH, USA) used at Human Anatomy and Embryology Department, Zagazig University.

Furthermore, the BAX to Bcl-2 ratio was determined for each sample individually by dividing the mean area percent for BAX by that for Bcl-2.

### Statistical analysis

Continuous variables were stated as the mean ± standard error of the mean (SEM) if the data were normally distributed. Normality was patterned by the Kolmogorov–Smirnov test. The one-way ANOVA was utilized to recognize significant changes between groups. Post hoc Tukey’s test was done for numerous comparisons among groups. Skewed continuous data were stated using the median and interquartile range (IQR). The Kruskal–Wallis test and Dunn’s multiple comparison test were used when equal variances were not present. The threshold for statistical significance was set at $p < 0.05$. All statistical computations were completed utilizing GraphPad Prism software, version 5.0 (GraphPad Software, San Diego, CA, USA).

## Results

### Biochemical results

#### Serum biochemical parameter levels of kidney function

All groups’ serum urea, uric acid, and creatinine levels are shown in Table 3. ALCL3 injection for 5 weeks significantly increased both serum urea and creatinine levels compared with the control and Hesperidin values ($p < 0.05$ vs control), which was more prominent in the creatinine level; however, uric acid level did not significantly change. Hesperidin co-treatment with ALCL3 injection further attenuated the changes in the level of serum markers of renal damage which revealed a significant decrement compared with the ALCL3 group, but a statistical equality with the control group was provided in serum urea ($p > 0.05$) only.

**Table 3:** Effect of ALCL3 and Hesperidin after ALCL3 injection for 5 weeks on kidney function of the different experimental groups

Parameters	Control	Hesperidin	ALCL3 group	ALCL3 + Hesperidin
Serum urea (mg/dL)	69.15 ± 2.38^a^	70.88 ± 2.68^a^	83.40 ± 0.91^b^	75.30 ± 0.58^c^
Serum uric acid (mg/dL)	2.11 ± 0.66^a^	2.13 ± 0.12^a^	2.677 ± 0.17^a^	2.302 ± 0.12^a^
Serum creatinine (mg/dL)	0.57 ± 0.03^a^	0.56 ± 0.03^a^	0.9285 ± 0.04^b^	0.6507 ± 0.06^b,^^c^

Values are represented as mean ± SEM

^a^Control and Hesperidin-treated groups

^b^Significantly different from the control and Hesperidin groups at$p < 0.05$

^c^Significantly different from the ALCL3 group at$p < 0.05$

#### Effects of ALCL3 and Hesperidin on proinflammatory cytokine IL-6 and anti-inflammatory cytokine IL-10

As shown in Table 4, rats treated with ALCL3 for 5 weeks exhibited significantly raised levels of cytokine IL-6. They showed decreased levels of cytokine IL-10 in renal tissue compared with rats of the control and Hesperidin groups. Renal tissue gained from rats co-treated with Hesperidin for 5 weeks with ALCL3 injection displayed significantly attenuated cytokine IL-6 levels and an increment in cytokine IL-10 levels when compared with the ALCL3 group rats. However, IL-6 levels still reveal a significant change when compared with the control group ($p < 0.05$).

**Table 4:** Effect of ALCL3 and Hesperidin after ALCL3 injection for 5 weeks on proinflammatory cytokine IL-6 and anti-inflammatory cytokine IL-10 in renal tissue homogenates of the different experimental groups

Parameters	Control	Hesperidin	ALCL3 group	ALCL3 + Hesperidin
Interleukin-6 (pg/gm tissue)	$106.4\pm 2.22$ ^a^	$106.0 \pm 3.36$ ^a^	$146.1 \pm 5.57$ ^b^	$126.2 \pm 3.19$ ^c^
Interleukin-10 (pg/gm tissue)	$104.3 \pm 1.63$ ^a^	$102.6 \pm 1.96$ ^a^	$80.62 \pm 1.53$ ^b^	$100.5 \pm 2.31$ ^c^

Values are represented as mean ± SEM

^a^Control and Hesperidin-treated groups

^b^Significantly different from the control and Hesperidin groups at$p < 0.05$

^c^Significantly different from the ALCL3 group at$p < 0.05$

#### Effect of ALCL3 and Hesperidin on oxidative stress markers in renal tissue

As presented in Table 5, lipid peroxidation, measured in terms of malondialdehyde (MDA), increased significantly in rat renal tissues treated with ALCL3 for 5 weeks compared with the control and Hesperidin groups ($p < 0.05$). The co-treatment with Hesperidin for 5 weeks with ALCL3 injection significantly decreased MDA concentration compared with the ALCL3 group ($p < 0.05$) but still revealed a significant change from the control and Hesperidin groups. Conversely, the TAC significantly decreased in the ALCL3 group compared with the control group ($p < 0.05$). However, the co-treatment with Hesperidin significantly increased TAC, with a non-significant difference from the control and Hesperidin groups.

**Table 5:** Effect of ALCL3 and Hesperidin after ALCL3 injection for 5 weeks on MDA level and total antioxidant capacity (TAC) in renal tissue homogenates of the different experimental groups

Parameters	Control	Hesperidin	ALCL3 group	ALCL3 + Hesperidin
MDA (nmol/mL-tissue)	$22.11 \pm 1.65$ ^a^	$21.98\pm 1.26$ ^a^	$50.99 \pm 1.24$ ^b^	$32.25 \pm 1.11$ ^bc^
Total antioxidant capacity (Mm-tissue)	$9.48 \pm 0.59$ ^a^	$10.04\pm 0.59$ ^a^	$5.46 \pm 0.64$ ^b^	$7.29 \pm 0.64$ ^c^

Values are represented as mean ± SEM

^a^Control and Hesperidin-treated groups

^b^Significantly different from the control and Hesperidin groups at$p < 0.05$

^c^Significantly different from the ALCL3 group at$p < 0.05$

### Effect of ALCL3 and Hesperidin on the gene expression of MMP-9

Real-time PCR was used to study the expression of MMP-9 mRNA. The mRNA expression of MMP-9 was significantly elevated ($p < 0.05$) in the ALCL3 group compared with the control and Hesperidin groups. The co-treatment with Hesperidin with the ALCL3 injection for 5 weeks revealed that the gene expression of MMP-9 was significantly reduced at the end of the experiment. There was a significant downregulation compared with ALCL3 ($p < 0.05$), as presented in Table 6.

**Table 6:** Effect of AlCl3 and Hesperidin on the gene expression of MMP-9

Parameters	Control	Hesperidin	ALCL3 group	ALCL3 + Hesperidin
Relative mRNA of MMP-9	$9.58 \pm 1.17$ ^a^	$9.24 \pm 0.8$ ^a^	$76.67 \pm 4.34$ ^b^	$24.17 \pm 2.12$ ^bc^

Values are represented as mean ± SEM

^a^Control and Hesperidin-treated groups

^b^Significantly different from the control and Hesperidin groups at$p < 0.05$

^c^Significantly different from the ALCL3 group at$p < 0.05$

### Histopathological results

#### Hematoxylin and eosin staining results

**Fig. 1:**
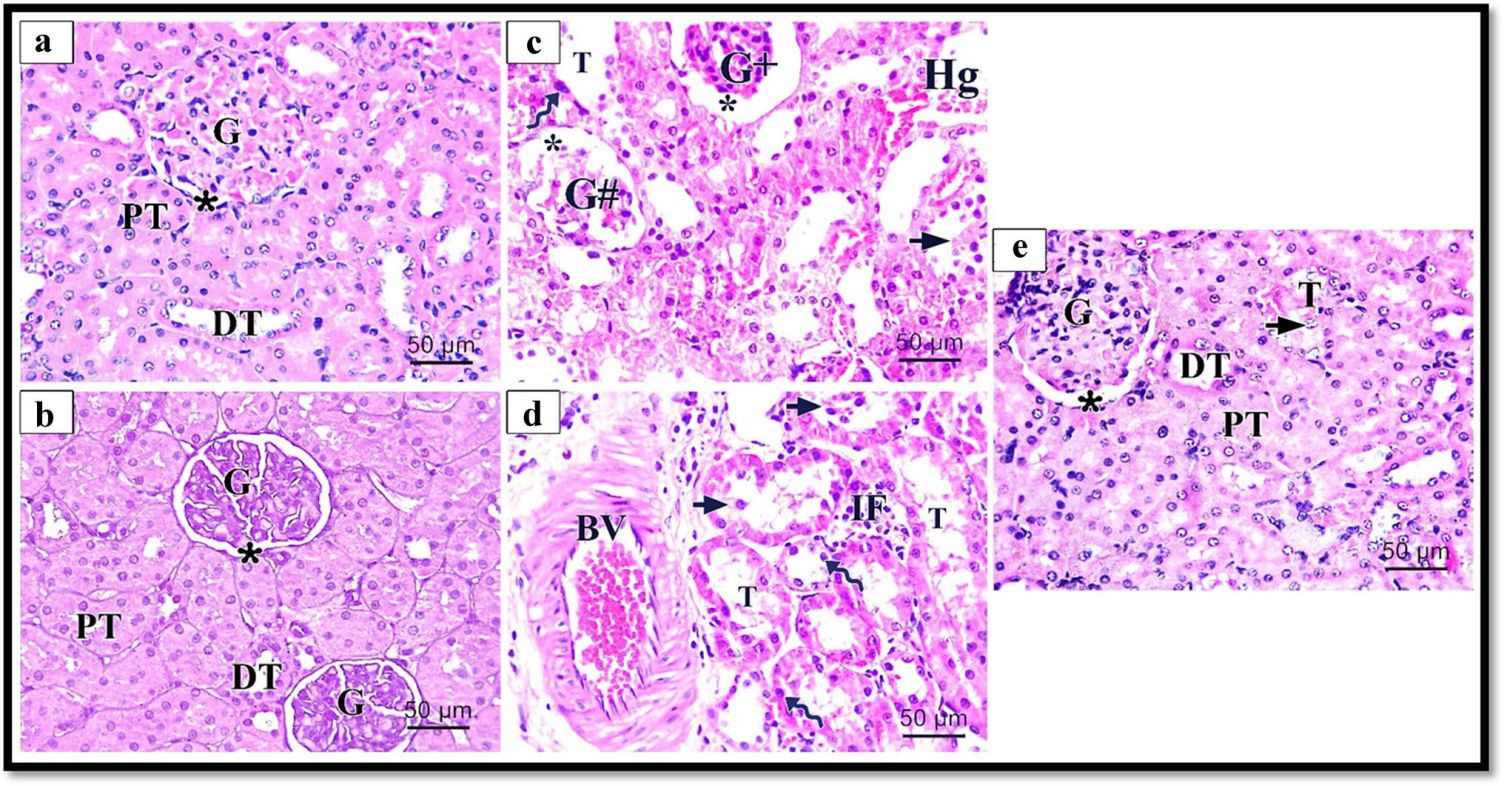
Illustrative images of rat renal cortex stained with H&E technique of different experimental groups. **a** The control group shows normal histological structure, glomeruli (G), proximal convoluted tubule (PT), distal convoluted tubule (DT), and Bowman’s space (asterisk). **b** The Hesperidin group shows normal histological structure, glomeruli (G), proximal convoluted tubule (PT), distal convoluted tubule (DT), and Bowman’s space (asterisk). **c, d** Rat’s renal cortex of the ALCL3-treated group shows different pathological lesions, shrunken glomerulus (G +), segmented glomerulus (G#), destructed tubules (T), interstitial hemorrhage (Hg), pyknotic nuclei (zigzag arrow), exfoliated epithelial lining (short arrow), large thick wall congested blood vessel (BV), interstitial cellular infiltrations (IF), and dilated Bowman’s space (asterisk). **e** Rat renal cortex of the ALCL3 + Hesperidin-treated group preserves their control character to a certain degree glomerulus (G), proximal convoluted tubule (PT), and distal convoluted tubule (DT). Few destructed tubules (T) show exfoliated epithelial lining (short arrow). H&E × 400, scale bar 50 μm

The rat renal cortex of the control and Hesperidin groups presented a typical histological construction of glomeruli with narrow renal glomerular capsular space. Proximal convoluted tubular cells with vesicular rounded basally situated nuclei and acidophilic granular cytoplasm and distal convoluted tubular cells were seen with less acidophilic cytoplasm (Fig. 1a, b) respectively. The rat renal cortex of groups treated with ALCL3 showed obvious pathological lesions, characterized by glomerular atrophy, and another segmented one with a wide renal glomerular capsular space, multiple renal tubular epithelial cell degeneration, and exfoliation in some tubules. Other tubules had pyknotic nuclei, renal interstitial hemorrhage, and interstitial cellular infiltration. Large, thick-walled congested blood vessels with irregular endothelial linings could be observed (Fig. 1c, d). The rats’ renal cortices of groups co-treated with Hesperidin with ALCL3 injection for 5 weeks displayed glomeruli. Proximal and distal convoluted tubular cells preserved their control character to a certain degree (Fig. 1e).

#### The EGTI histopathological scoring system

The scoring system consisted of histological injury in 4 individual components: Endothelial, Glomerular, Tubular, and Interstitial. The scoring was made in all studied groups of the renal cortex after histopathological examination by light and electron microscopy. The control rats exhibited a normal appearance of the renal cortex; the brush border of the tubular cells is intact, with no thickening of the basal membrane. No inflammation or necrosis was seen (Tubular score 0). There is no noticeable interstitium suggesting no damage/abnormality within the interstitial compartment (Interstitial score 0). The ALCL3 group showed varying degrees of damage to the renal cortex, inflammation, and hemorrhage within the interstitium. It presented in less than 25% of the tissue with thickened basal membranes of the tubular cells with loss of the brush border in more than 25% of the tubular cells, with necrosis (Tubular score 3, $p < 0.05$) compared with the control and Hesperidin groups.

Additionally, there were inflammation and hemorrhage within the interstitial space, with necrosis in up to 60% of the cells (Interstitial score 3). In the ALCL3 + Hesperidin group, the brush border of the tubular cells is intact, with a mild thickening of the basal membrane. Few inflammations with absent necrosis appeared (Tubular score 1). There is no noticeable interstitium, suggesting no injury within the interstitial compartment (Interstitial score 0, $p < 0.05$), compared with the aluminum chloride-treated group (Table 7).

**Table 7:** The EGTI histology scoring results

EGTI damage score	Control	Hesperidin	AlCl3	AlCl3 + Hesperidin
Endothelial (median (IQR))	0.0 (0.25)^a^	0.0 (0.25)^a^	3.0 (0.0)^b^	0.5 (1.25)^bc^
Glomerular (median (IQR))	0.0 (0.25)^a^	0.0 (0.25)^a^	3.0 (1.0)^b^	1.0 (1.25)^bc^
Tubular (median (IQR))	0.0 (1.0)^a^	0.0 (1.25)^a^	4.0 (1.0)^b^	1.0 (2.0)^bc^
Interstitial (median (IQR))	0.0 (1.0)^a^	0.0 (1.25)^a^	3.0 (1.25)^b^	1.0 (2.0)^bc^

^a^Control and Hesperidin-treated groups

^b^Significantly different from the control and Hesperidin groups at$p < 0.05$

^c^Significantly different from the ALCL3 group at$p < 0.05$

#### Masson’s trichrome staining results

**Fig. 2:**
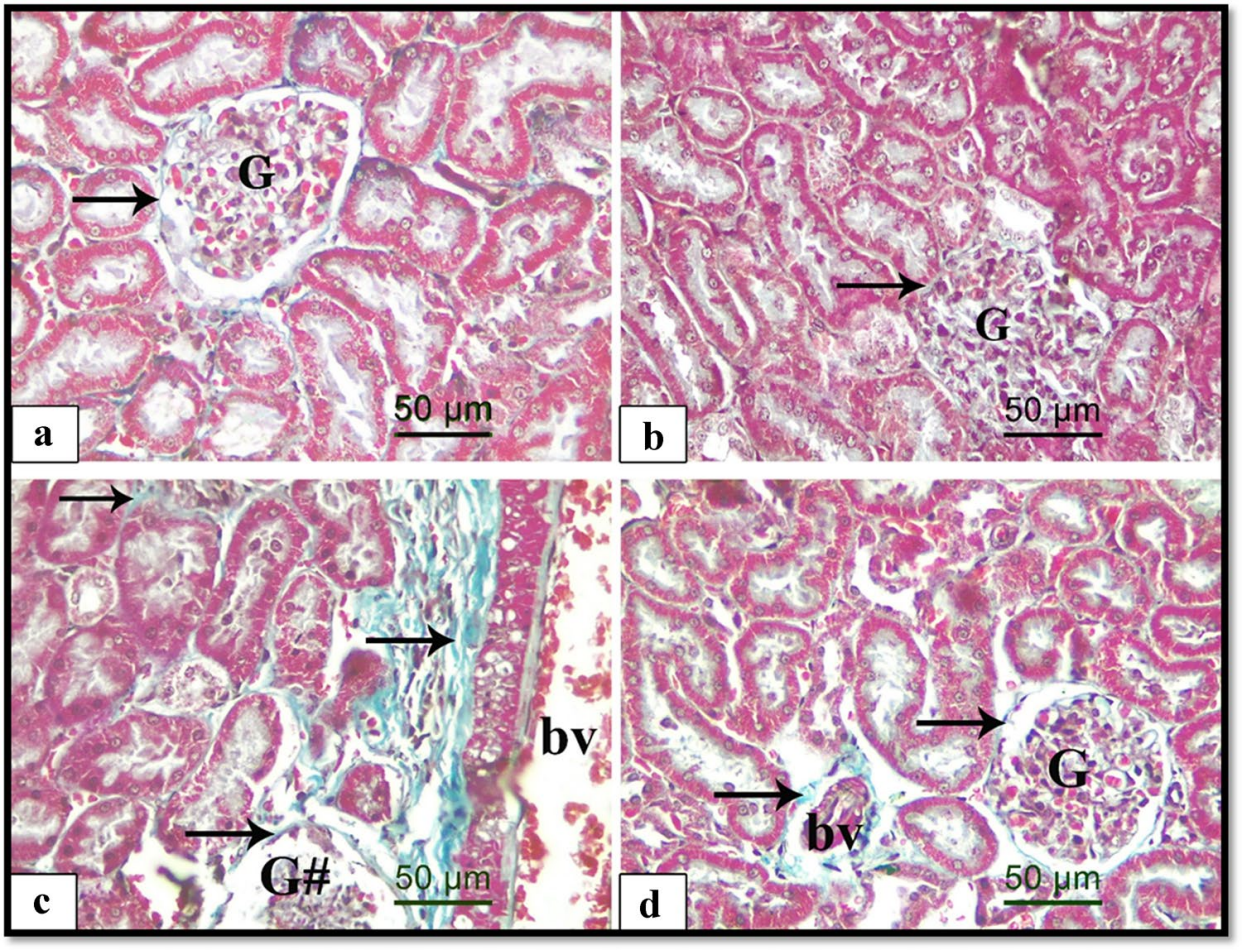
Illustrative images of Masson’s trichrome-stained sections of different experimental groups showing the collagen fiber distribution. **a** Control group, **b** Hesperidin group, **c** ALCL3, and **d** ALCL3 + Hesperidin groups. Arrows indicating green staining of the collagen fibers in the interstitium and around normal glomeruli (G), destructive ones (G#), and the blood vessels (bv). Masson’s trichrome, scale bar × 50 μm, × 400

The results obtained by Masson’s trichrome staining of all experimental groups are presented in Fig. 2. The control and Hesperidin groups showed minimal basophilic collagen fibers adjacent to the glomeruli (Fig. 2a, b). In contrast, the ALCL3-treated group exhibited abundant collagen fibers surrounding the renal tubules and around the blood vessels (Fig. 2c). However, the concomitant administration of Hesperidin with ALCL3 exhibited few collagen fibers around the glomeruli and the blood vessels (Fig. 2d). These findings were confirmed through the statistical analyses of the area percentage of collagen fibers in all experiment groups (Table 8).

**Table 8:** Statistical analysis of the histomorphometric measurements

Morphometric measurements	Control	Hesperidin	ALCL3 group	ALCL3 + Hesperidin
Area percent of Masson’s trichrome	$5.2 \pm 0.39$ ^a^	$5.04 \pm 0.29$ ^a^	12.88 ± 0.29^b^	7.87 ± 0.39^bc^
Optical density of MMP-9	0.2 ± 0.01^a^	0.22 ± 0.01^a^	0.39 ± 0.002^b^	0.31 ± 0.002^bc^
Area percent of FAS	$2.87 \pm 0.5$ ^a^	$2.77 \pm 0.47$ ^a^	$17.33\pm 0.82$ ^b^	$8.83\pm 0.84$ ^bc^
Area percent of caspase-3	$0.32 \pm 0.039$ ^a^	$1.73 \pm 0.13$ ^a^	$20.23 \pm 0.6$ ^b^	$7.1 \pm 0.37$ ^bc^
Area percent of BAX	$1.92 \pm 0.17$ ^a^	$1.91 \pm 0.24$ ^a^	$28.02\pm 1.36$ ^b^	$8.01\pm 0.9$ ^bc^
Area percent of BCL2	$25.49 \pm 1.53$ ^a^	$28.99\pm 2.26$ ^a^	$3.61\pm 0.24$ ^b^	$17.91\pm 1.96$ ^bc^

Values are represented as mean ± SEM

^a^Control and Hesperidin-treated groups

^b^Significantly different from the control and Hesperidin groups at$p < 0.05$

^c^Significantly different from the ALCL3 group at$p < 0.05$

#### Immune histochemical results

**Fig. 3:**
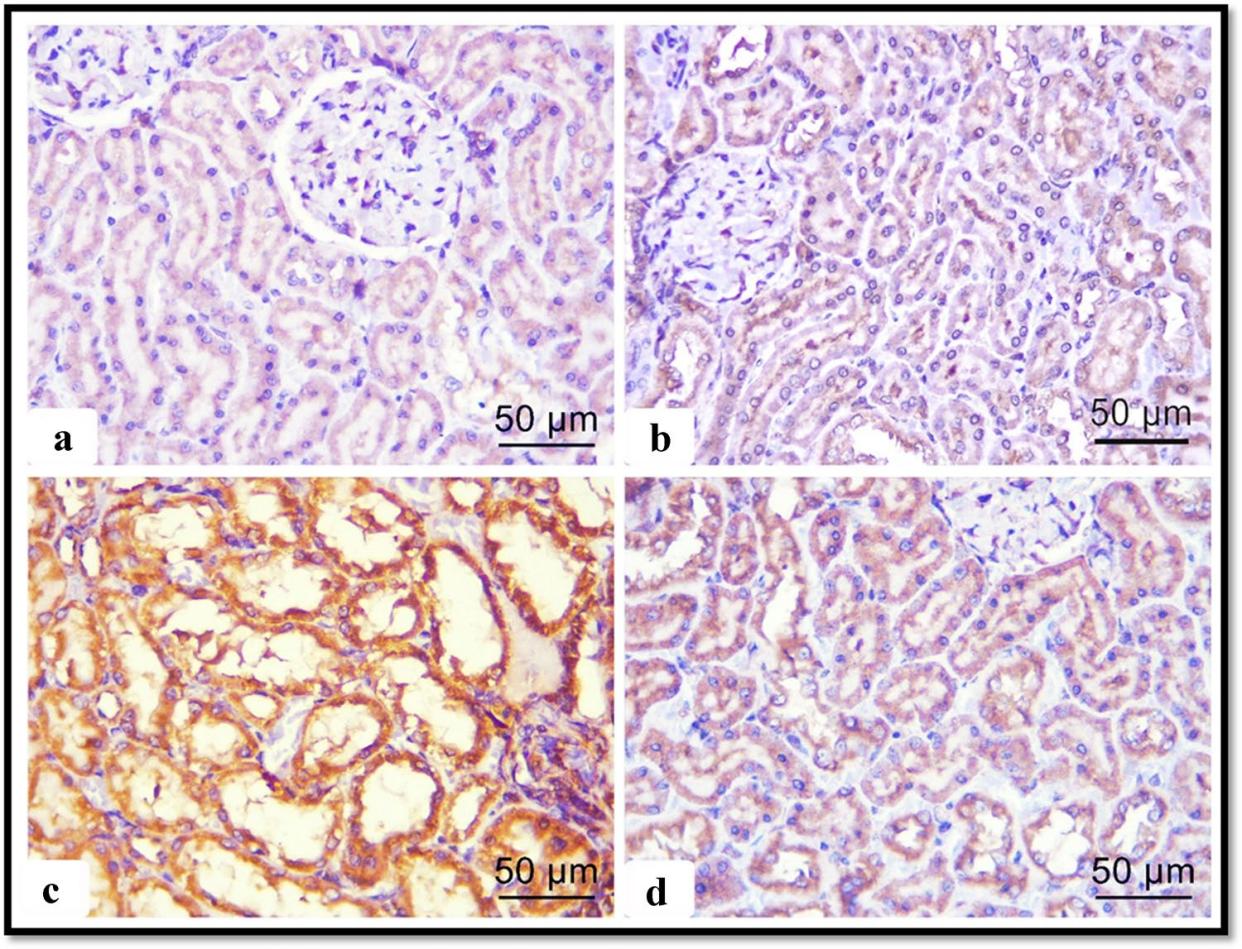
Illustrative images of sections of rat renal cortex showing matrix metalloproteinase-9 (MMP-9) immunoreactivity in the different experimental groups: **a** control, **b** Hesperidin, **c** ALCL3-treated, and **d** ALCL3 + Hesperidin groups. The dark brown expression indicates the MMP-9 immunopositivity. Immunoperoxidase technique for MMP-9, × 50 μm, × 400

To explore the effects of ALCL3 and Hesperidin on the oxidation process, we measured the matrix metalloproteinase-9 (MMP-9) as an oxidative and fibrotic marker. Immunoexpression in the renal specimens of all groups was analyzed. Immunohistochemically stained renal sections of the control and Hesperidin groups exhibited faint positive reactions for MMP-9 in the cytoplasm of tubular cells (Fig. 3a, b), respectively. Strong positive reactions for MMP-9 in the cytoplasm of degenerated tubular cells appeared in the ALCL3 group (Fig. 3c). The ALCL3 + Hesperidin group revealed weak positive reactions for MMP-9 in the cytoplasm of most tubular cells compared with the treated group (Fig. 3d). The outcomes confirmed statistically in the ALCL3-treated group displayed a significant increase in the optical density (OD) of the positive immune appearance of the MMP-9 compared with the control and Hesperidin groups. These results were significantly lower in rats receiving ALCL3 and Hesperidin together ($p < 0.05$) (Table 8).

**Fig. 4:**
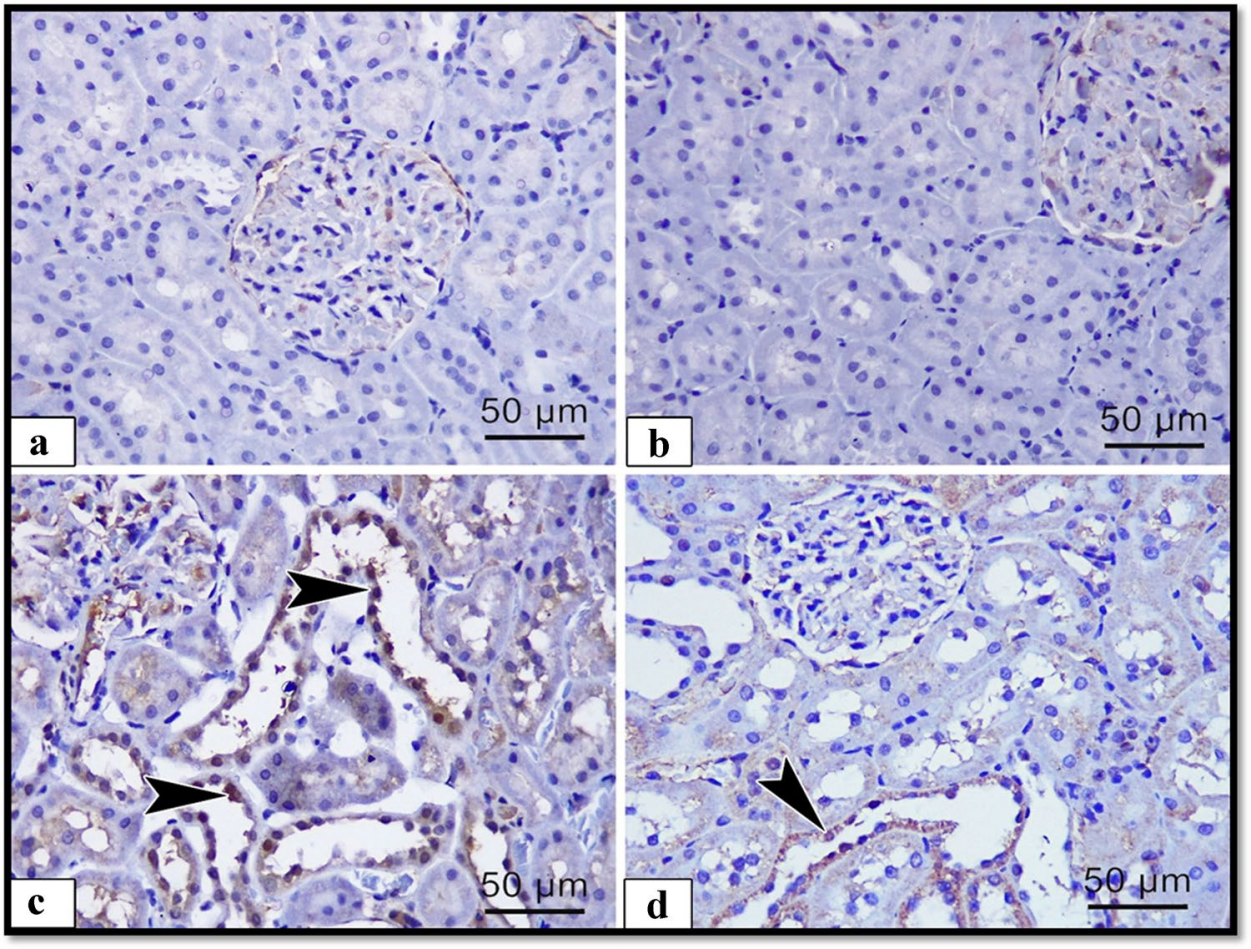
Illustrative images of sections of rat renal cortex showing FAS immunoreactivity in the different experimental groups: **a** control group, **b** Hesperidin, **c** ALCL3, and **d** ALCL3 + Hesperidin groups, respectively. Arrow heads are signifying the dark brown expression of FAS-positive cells. Immunoperoxidase technique for FAS, × 50 μm, × 400

**Fig. 5:**
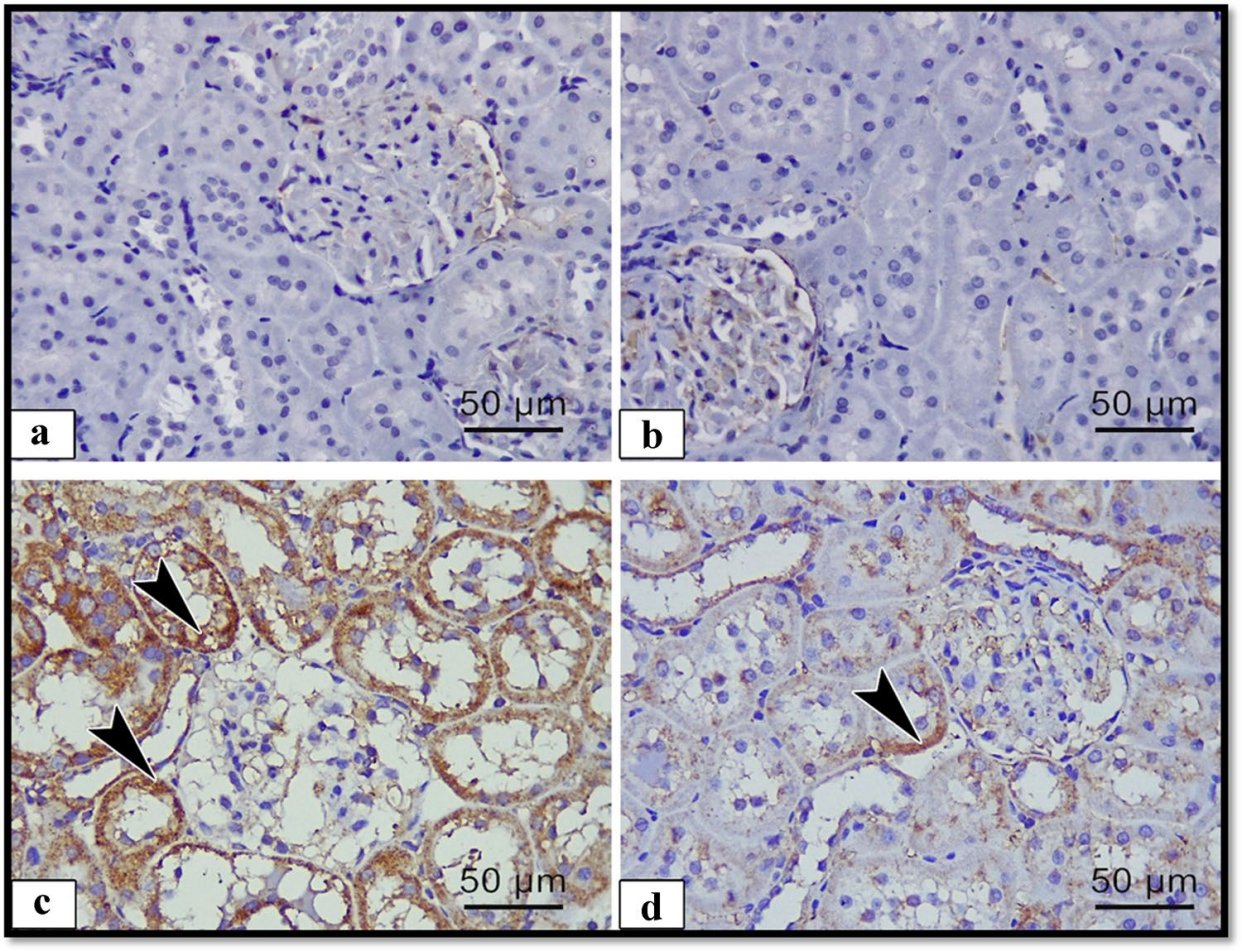
Illustrative images of sections of rat renal cortex showing caspase-3 immunoreactivity in the different experimental groups: **a** control, **b** Hesperidin, **c** ALCL3, and **d** ALCL3 + Hesperidin groups, respectively. Arrow heads are signifying the dark brown expression of caspase-3-positive cells. Immunoperoxidase technique for caspase-3, × 50 μm, × 400

**Fig. 6:**
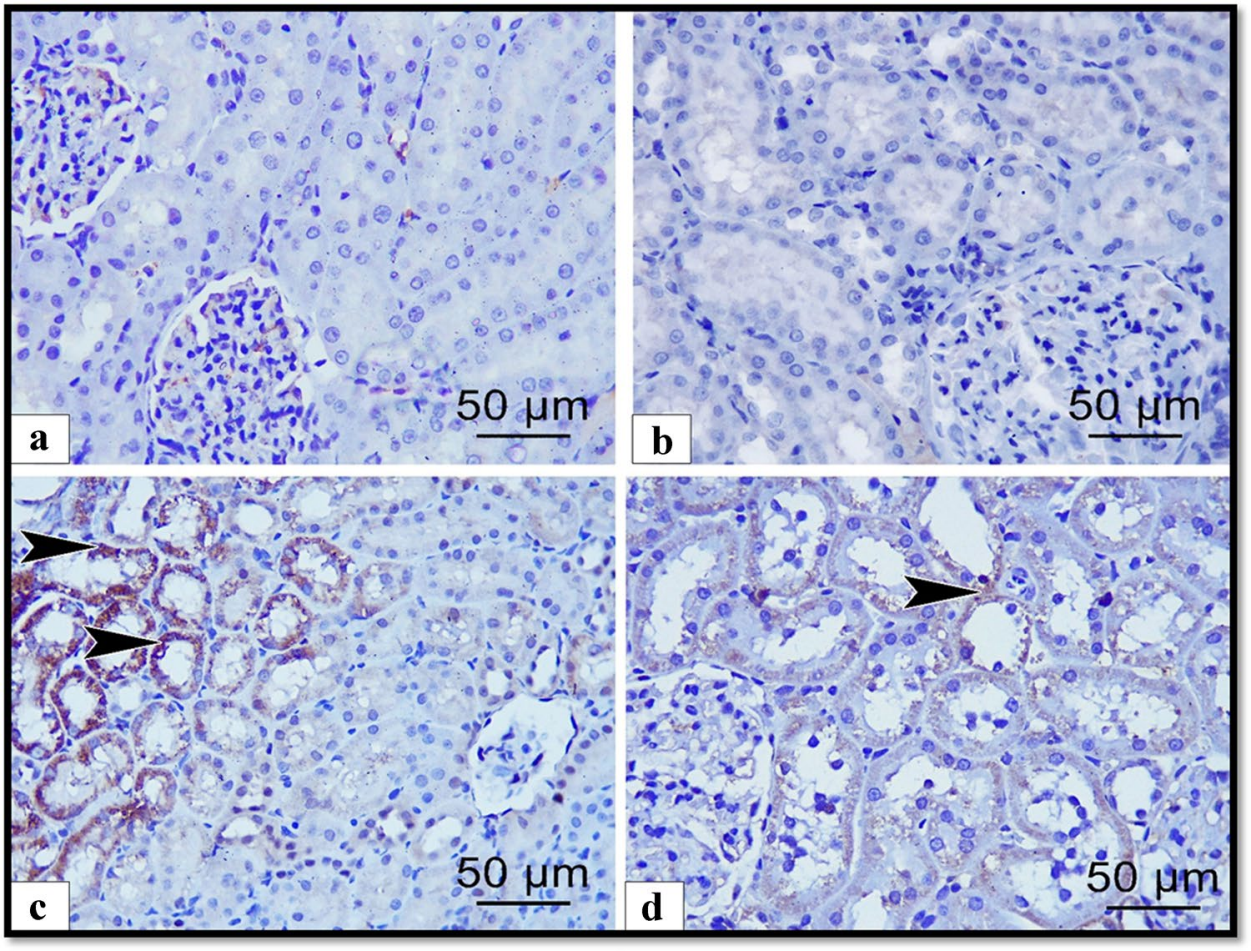
Illustrative images of sections of rat renal cortex showing BAX immunoreactivity in the different experimental groups: **a** control, **b** Hesperidin, **c** ALCL3, and **d** ALCL3 + Hesperidin groups, respectively. Arrow heads are signifying the dark brown expression of BAX-positive cells. Immunoperoxidase technique for BAX, × 50 μm, × 400

**Fig. 7:**
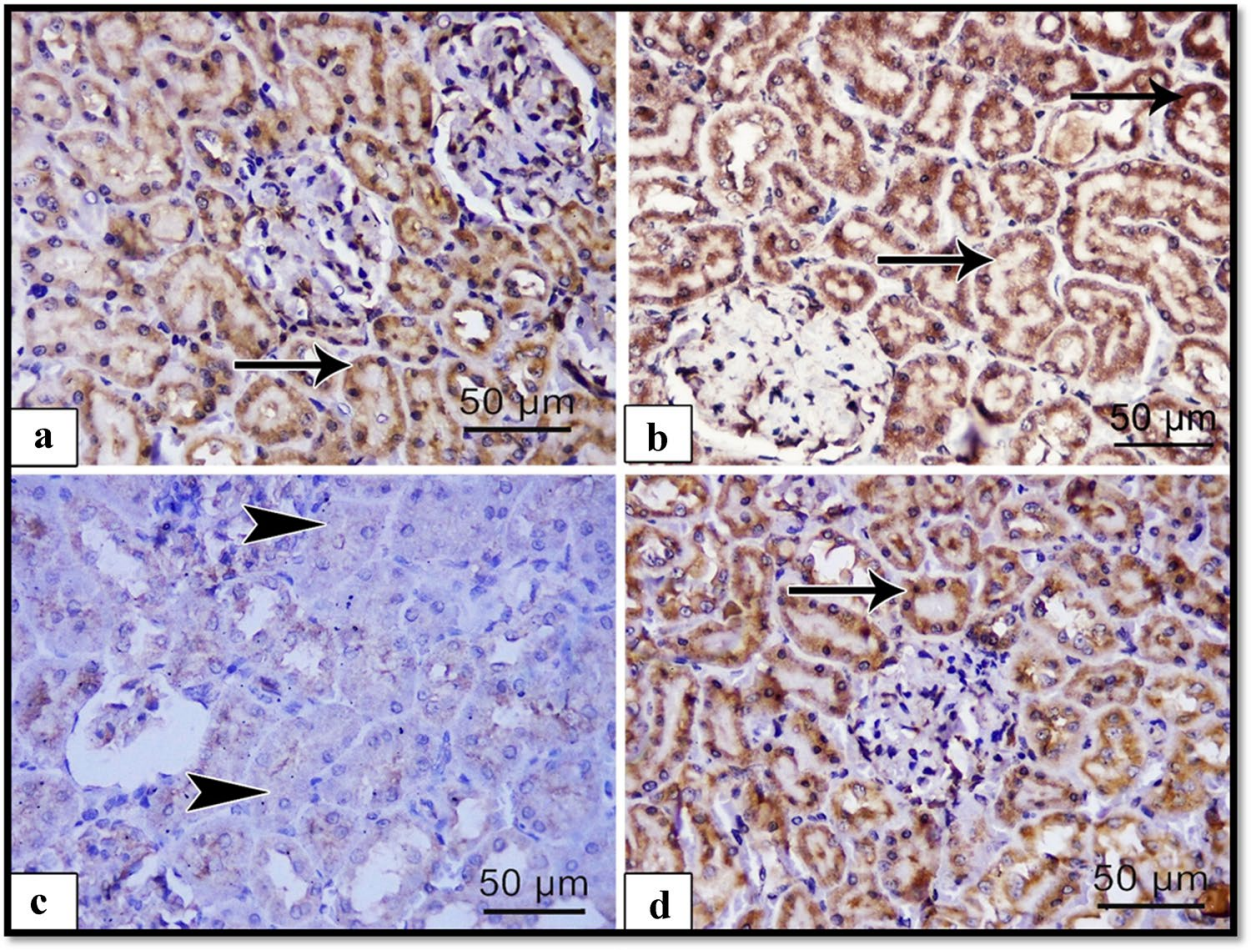
Illustrative images of sections of rat renal cortex showing BCL2 immunoreactivity in the different experimental groups: **a** control, **b** Hesperidin, **c** ALCL3, and **d** ALCL3 + Hesperidin groups, respectively. Arrows are signifying the markedly expressed reaction of BCL2-positive cells. Arrow heads are corresponding to the hardly demonstrated BCL2-positive cells. Immunoperoxidase technique for BCL2, × 50 μm, × 400

To detect the effect of ALCL3 and Hesperidin on cell survival, we measured FAS, caspase-3, and BAX proteins as apoptotic markers and Bcl2 as antiapoptotic marker. Immunoexpression in the renal samples was analyzed (Figs. 4, 5, 6, and 7), respectively. In the renal tubules of the control and Hesperidin groups, FAS, caspase-3, and BAX-positive apoptotic cells were hardly demonstrated (Figs. 4a, b, 5a, b, and 6a, b), respectively, and Bcl2-positive cells were markedly expressed in the renal tissue (Fig. 7a, b). In the ALCL3-treated group, abundant FAS, caspase-3, and BAX-positive apoptotic cells were demonstrated in the renal tubules (Figs. 4c, 5c, and 6c), respectively, and Bcl2-positive cells were hardly demonstrated in the renal tubules (Fig. 7c). On the other hand, the use of Hesperidin in concomitant with ALCL3 greatly decreased the FAS, caspase-3, and BAX expression that exhibited relatively fewer apoptotic cells in the renal tissues (Figs. 4d, 5d, and 6d), respectively, and greatly increased the Bcl2 expression in the renal tissues (Fig. 7d). These results confirmed statistically that the ALCL3-treated group exhibited a significant increase in the area percentage of the immune-positive appearance of the FAS, caspase-3, and BAX and a significant decrease in the area percentage of the immune-positive appearance of the Bcl2 compared with the control group.

Interestingly, Hesperidin co-administration with ALCL3 showed antiapoptotic effect as it significantly decreased BAX and increased BCL-2 immune-expression area percent at ($p < 0.05$) as compared to the ALCL3-treated group (Table 8).

Furthermore, by BAX/BCL2 protein ratio application, there was a statistically significant increase in the ALCL3-treated group in comparison with the control and Hesperidin-treated groups with statistically significant decrease in the ALCL3 + Hesperidin group ($p < 0.05$) (Table 9).

**Table 9:** The BAX to Bcl-2 protein ratio

Morphometric measurements	Control	Hesperidin	ALCL3 group	ALCL3 + Hesperidin
The BAX to Bcl-2 ratio	0.075^a^	0.065^a^	7.76^b^	0.44^bc^

^a^Control and Hesperidin-treated groups

^b^Significantly different from the control and Hesperidin groups at$p < 0.05$

^c^Significantly different from the ALCL3 group at$p < 0.05$

### Semi-thin toluidine blue-stained sections

**Fig. 8:**
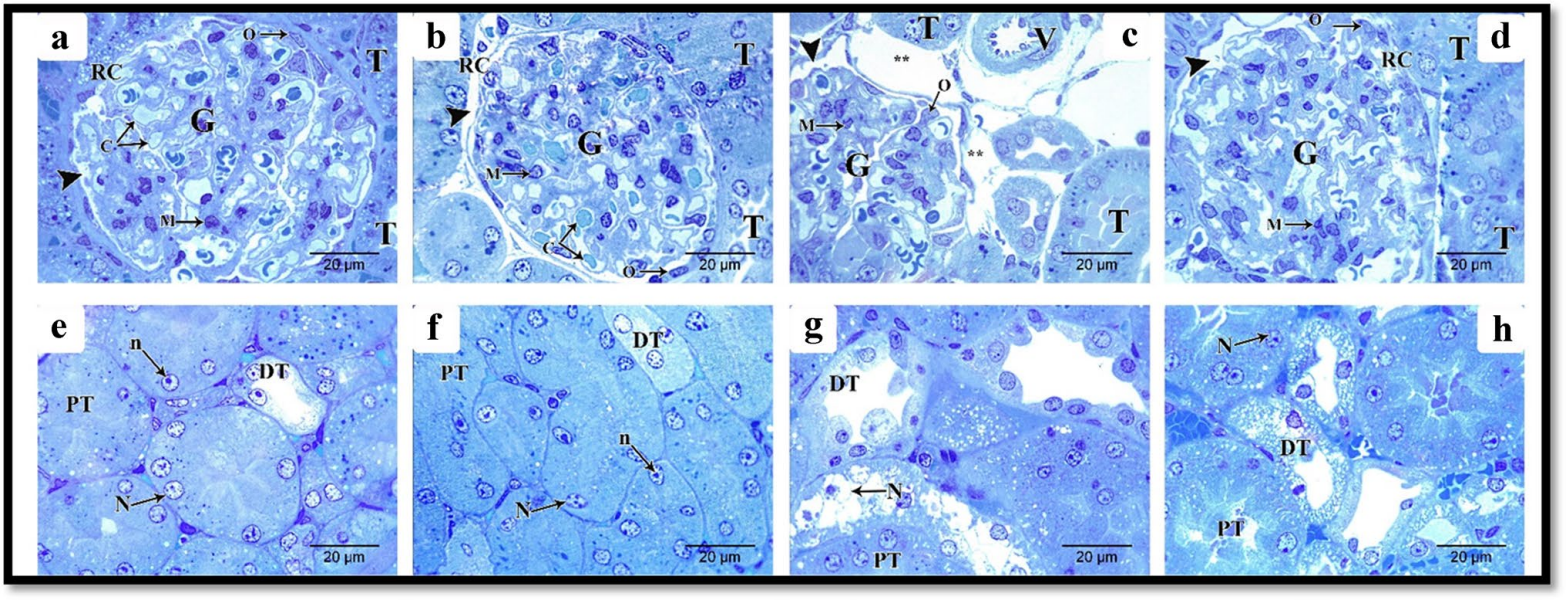
Semi-thin sections in the rat renal cortex from the different experimental groups. **a**, **e**, **b**, **f** Control and Hesperidin groups, respectively. **a**, **b** Tubules (T) and a part of the renal corpuscle (RC) is lined by parietal layer of Bowman’s capsule (arrowhead). The podocytes (O), which line the visceral layer, embrace the glomerular capillaries (C) and mesangial cells (M) in both control and Hesperidin groups, respectively. **e**, **f** Proximal convoluted tubule has narrow lumen vesicular nuclei (N) with nucleoli (n) and basal striations (PT), distal tubules have cuboidal epithelium with rounded vesicular nuclei and basal striation (DT) in both control and Hesperidin groups, respectively. **c**, **g** ALCL3 group. **c** The renal corpuscle exhibits narrowed renal space (arrowhead), but there are dilated inter-tubular capillaries (**) in the interstitium with thick wall blood vessels (v). **g** Some nuclei of proximal (PT) and distal (DT) tubular cells appear dense and distorted (N). Renal tubules have vacuolated cytoplasm with loss of the cellular architecture. **d, h** ALCL3 + Hesperidin group. **d** The renal corpuscles (RC) with preserved architecture and capsular thickness and space (arrowhead). The podocytes (O) embrace mesangial cells (M). **h** Notable restoration of the brush border and the vesicular nuclei (N) in both proximal (PT) and distal convoluted tubules (DT). Toluidine blue, × 20 μm, × 1000

Examination of the semi-sections stained by toluidine blue of renal cortex from the different experimental groups is shown in Fig. 8. In the control and Hesperidin groups, the renal corpuscle was lined by the parietal layer of Bowman’s capsule. The podocytes, which lined the visceral layer, embraced the glomerular capillaries and mesangial cells (Fig. 8a, b), respectively. The proximal convoluted tubule had narrow lumen, vesicular nuclei with nucleoli, and basal striations. The distal tubules had cuboidal epithelia with rounded vesicular nuclei and basal striations (Fig. 8e, f), respectively. In the ALCL3 group, the renal corpuscle exhibited a narrowed renal space; however, there were dilated inter-tubular capillaries in the interstitium with thick-walled blood vessels. Some nuclei of tubular cells appeared dense and distorted, whereas others exhibited rarified chromatin (Fig. 8c). Renal tubules had vacuolated cytoplasm with loss of the cellular architecture (Fig. 8g). In the ALCL3 + Hesperidin group, the renal corpuscles had preserved architecture and capsular thickness and space (Fig. 8d). There was a notable restoration of the brush border and the vesicular nuclei in the proximal and distal convoluted tubules (Fig. 8h).

### Ultrastructural results

#### Podocytes, glomerular capillaries, and glomerular basement membrane

**Fig. 9:**
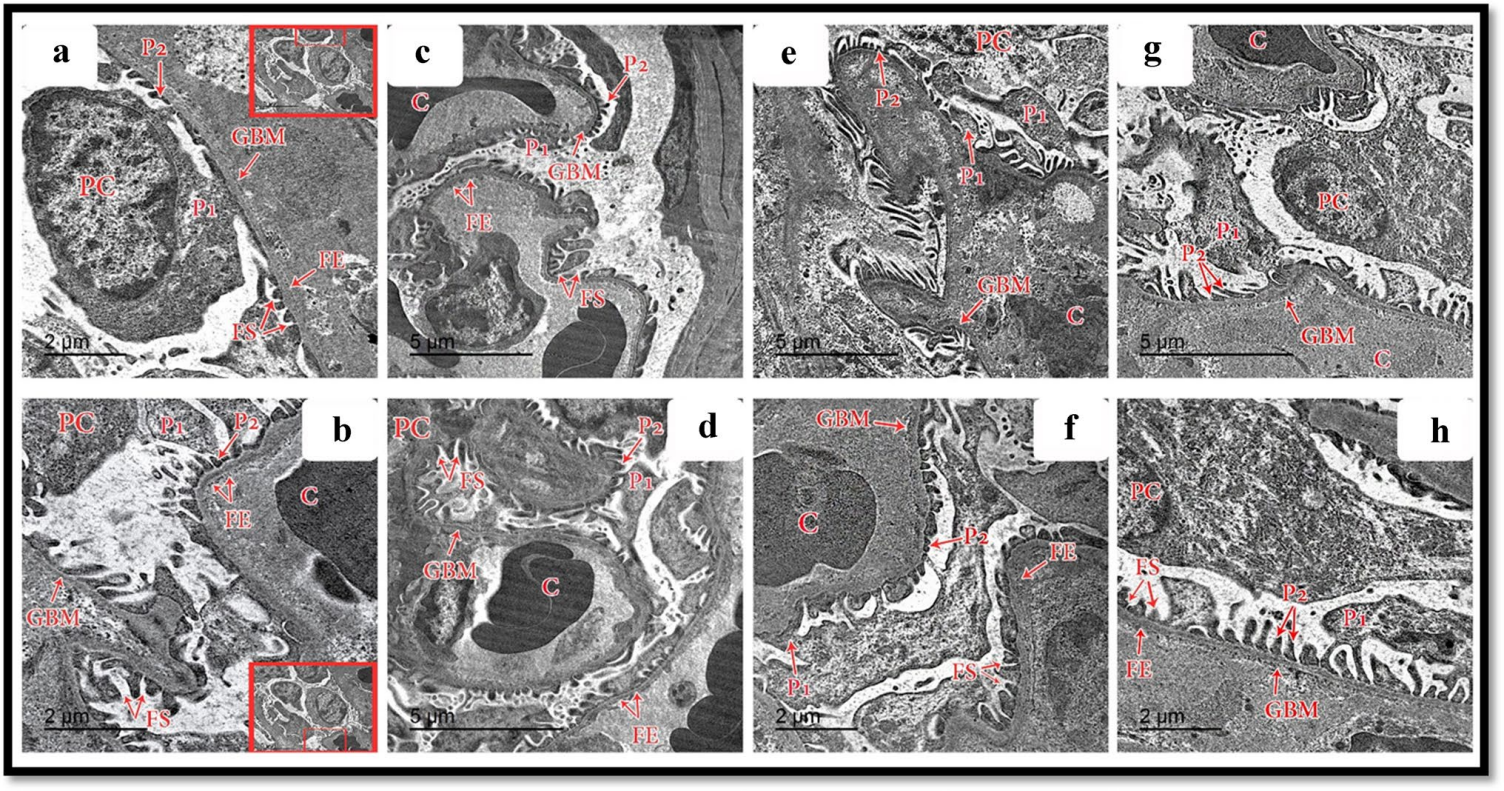
Transmission electron microscopy (TEM) of the renal corpuscle of rat renal cortex showing a panoramic view of a glomerulus from the different experimental groups. **a**, **b** The control group shows a higher magnification of the right upper and lower corners of TEM, respectively, showing podocytes (PC) with well-defined intact foot processes primary (P1) and secondary (P2), glomerular capillaries (C), intact basement membrane (GBM), fenestrated endothelium (FE), filtration slits (FS). **c**, **d** The Hesperidin group shows podocytes (PC) with well-defined intact foot processes primary (P1) and secondary (P2), glomerular capillaries (C), intact basement membrane (GBM), fenestrated endothelium (FE), filtration slits (FS). **e**, **f** The ALCL3 group shows podocytes (PC) with dark-stained nuclei and fused and ill-defined or even disappeared primary (P1) and secondary (P2) foot processes, congested glomerular capillaries (C), thickened basement membrane (GBM), fenestrated endothelium (FE), filtration slits (FS). **g**, **h** The ALCL3 + Hesperidin group shows podocytes (PC), with partially intact primary (P1) and secondary (P2) foot processes, glomerular capillaries (C), partially thickened basement membrane (GBM), fenestrated endothelium (FE), filtration slits (FS). TEM: **a**, **b**, **f**, **h**: scale bar = 2 μm; **c**, **d**, **e**, **g**: scale bar = 5 μm

Transmission electron microscopic assessment of the ultrathin section of the control and Hesperidin groups documented that the glomerular capillaries had fenestrated endothelial lining surrounded by the glomerular basement membrane. Podocytes showed folded nuclei and major processes (primary processes) extending parallel to the glomerular basement membrane (GBM). Numerous minor processes extended from the major ones (secondary processes) and passed perpendicularly to the GBM to end by feet-like plates separated by filtration slits in both control and Hesperidin groups (Fig. 9a, c), respectively. The glomerular filtration barrier was formed by three layers: fenestrated capillary endothelium, trilaminar GBM, and filtration slits between the minor processes of podocytes in both control and Hesperidin groups (Fig. 9b, d), respectively. In the ALCL3 group, TEM results of the renal cortex showed an irregular thickening of the GBM with the loss of its trilaminar appearance (Fig. 9e). In addition, the minor processes of podocytes appeared enlarged and fused, obliterating the filtration slits (Fig. 9f). In the ALCL3 + Hesperidin group, the GBM appeared to be mostly of regular thickness, except for some thickened areas (Fig. 9g). Filtration slits separated the minor processes of the podocytes. The glomerular capillaries were lined by fenestrated endothelium with partially intact primary and secondary foot processes (Fig. 9h).

#### Proximal convoluted tubules

**Fig. 10:**
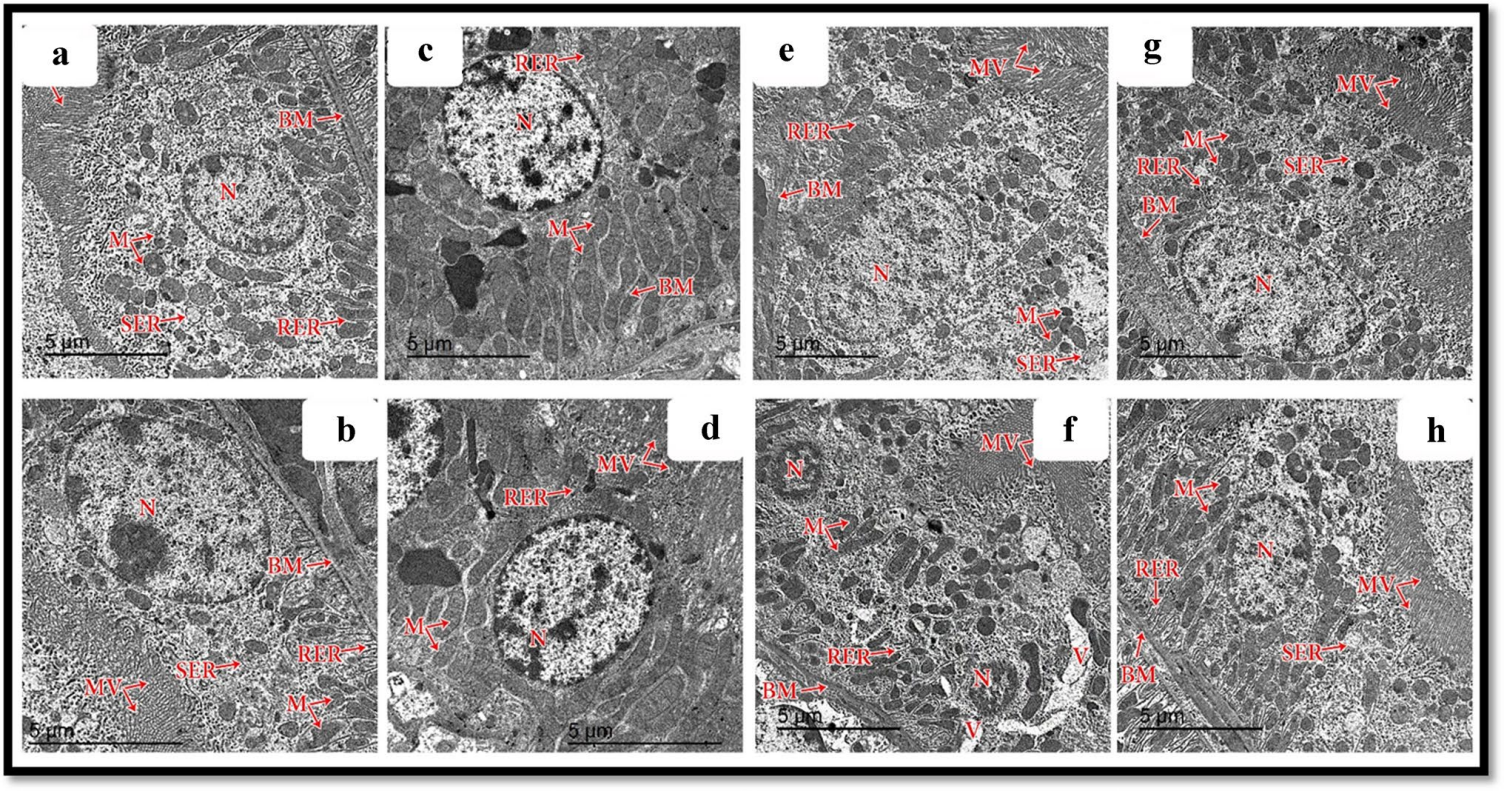
Transmission electron microscopy (TEM) of the proximal convoluted tubules (PCT) of rat renal cortex from the different experimental groups. **a**, **b**, **c**, **d** The control and Hesperidin groups, respectively, show PCT cells with euchromatic nuclei (N), resting on a thin intact basement membrane (BM). Their apical borders demonstrated numerous closely packed microvilli (MV). In addition, numerous tubular longitudinally arranged mitochondria (M), well-developed rough (RER) and smooth (SER) endoplasmic reticula are seen. **e**, **f** The ALCL3 group shows lining cells of PCTs with cytoplasmic vacuoles (V), increased density of the nucleus (N), thickened basement membrane (BM), loss of mitochondrial (M) longitudinal arrangement, hypertrophied RER and SER, and disturbed appearance of the apical microvilli (MV). **g**, **h** The ALCL3 + Hesperidin group shows the cells lining PCTs have euchromatic central nuclei (N) resting on a moderately thin intact basement membrane (BM), nearly normal apical microvilli (MV) nearly preserved RER and SER, and preserved mitochondrial (M) longitudinal arrangement. TEM, scale bar = 5 μm

The cells lining the PCTs appeared cuboidal in the control and Hesperidin groups, with euchromatic central nuclei resting on a thin intact basement membrane. Their apical borders demonstrated numerous closely packed, well-developed microvilli projecting into the lumen. Also, numerous tubules, longitudinally arranged mitochondria, and rough and smooth endoplasmic reticula were seen in both control and Hesperidin groups (Fig. 10a–d), respectively. In the ALCL3 group, the lining cells of PCTs showed cytoplasmic vacuoles, increased density of the nuclei, thickened basement membranes, loss of mitochondrial longitudinal arrangement, hypertrophied RER and SER, and distorted appearances of the apical microvilli (Fig. 10e, f). Regarding the ALCL3 + Hesperidin group, the cells lining the PCTs had euchromatic central nuclei resting on a moderately thin, intact basement membrane, intact apical microvilli, and a preserved mitochondrial longitudinal arrangement (Fig. 10g, h).

#### Distal convoluted tubules

**Fig. 11:**
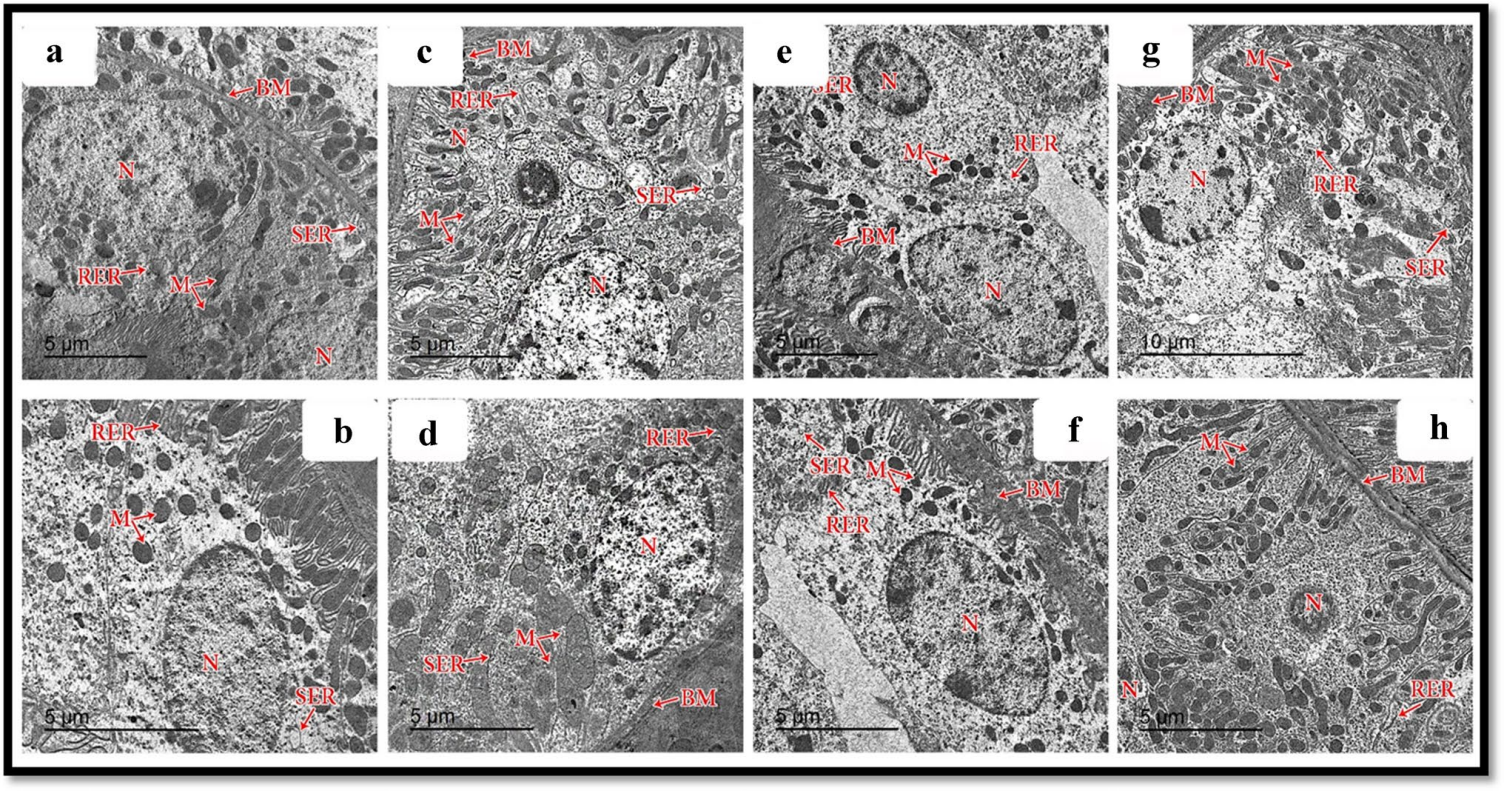
Transmission electron microscopy (TEM) of the distal convoluted tubules (DCT) of rat renal cortex from the different experimental groups. **a**, **b**, **c**, **d** The control and Hesperidin groups, respectively, show DCT cells with euchromatic nuclei (N), resting on a thin intact basement membrane (BM). In addition, basally arranged mitochondria (M), well-developed rough (RER) and smooth (SER) endoplasmic reticula are seen. **e**, **f** The ALCL3 group shows lining cells of DCTs with widening of the intercellular spaces and irregular nuclear outlines (N), thickened basement membrane (BM), loss of mitochondrial (M) basal arrangement, hypertrophied RER and SER. **g**, **h** The ALCL3 + Hesperidin group shows the cells lining DCTs had euchromatic central nuclei (N) resting on a moderately thin intact basement membrane (BM), nearly preserved RER and SER, and mild disarrangement of mitochondria (M). TEM: **a**–**h**: scale bar = 5 μm; **g**: scale bar = 10 μm

The cubical cells lining the DCTs had few short-scattered microvilli on their luminal surfaces in the control and Hesperidin groups. Each cell of the DCT contained an ovoid euchromatic nucleus with less extended chromatin resting on a thin, intact basement membrane. Furthermore, basally arranged mitochondria and well-developed rough and smooth endoplasmic reticula occurred in both control and Hesperidin groups (Fig. 11a–d), respectively. In the ALCL3 group, the lining cells of the DCTs showed widening of the intercellular spaces and irregular nuclear outlines, together with the loss of the basal arrangement of its mitochondria and thickened basement membrane with hypertrophied (RER) and (SER) (Fig. 11e, f). Regarding the ALCL3 + Hesperidin group, the cells lining the DCTs had moderately thin, intact basement membranes, nearly preserved RER and SER, and a mild disarrangement of the mitochondria (Fig. 11g, h).

## Discussion

Nowadays, the extreme exposure to aluminum due to dissimilar human daily lifestyles raised the threat of renal aluminum withholding due to the accumulation of aluminum to the renal tubules which resulted in renal dysfunction as reported by Al Dera (2016) and Hasona and Ahmed (2017).

The present study has provided a more direct investigation of the precise role of ALCL3 in the incidence of renal dysfunction. The study aimed to investigate the potential molecular mechanisms of Hesperidin-induced nephroprotection in ALCL3-induced renal injury. We utilized 6-week-old age rats as it is the age of rat maturity according to the international experimental guidelines (ARRIVE guidelines) (Percie du Sert et al. 2020). We administered ALCL3 in a dose of 10 mg /kg as documented by (Mostafa et al. 2015), but we decided to increase the duration from 4 to 5 weeks to ensure the occurrence of the renal injury at all levels.

Our findings accounted for the reported disturbance in plasma level of creatinine, urea, and uric acid as evidenced by significant elevated level of serum creatinine and urea as well as uric acid levels as compared to the control and Hesperidin groups which were assessed as significant indicators of renal function and increased their levels after ALCL3 administration, indicating impaired renal function and provoking nephrotoxicity. Such outcomes were consistent with the previous literature reported results by Al-Kahtani and Morsy (2019), Balgoon (2019), and Okail et al. (2020). Additionally, Imam et al. (2016) informed that the raised plasma urea and creatinine readings in ALCL3-treated rats are reflected as a considerable marker of renal dysfunction.

Moreover, in the current study, a noticeably enhanced intrarenal oxidative stress with significant elevation in renal MDA level (the byproduct of lipid peroxidation) as compared to the control and Hesperidin groups and overwhelming defensive antioxidant molecules proved by the diminished TAC of the renal tissue against the harm of ROS following ALCL3 administration. Such outcomes are consistent with those of Tribble et al. (1987) and Pari et al. (2015).

Furthermore, ALCL3 significantly elevated proinflammatory cytokine levels (including IL-6) and decreased anti-inflammatory markers such as IL-10, which is consistent with many previous reports that revealed significant elevations in proinflammatory cytokine levels (including TNF-α and IL-6) in ALCL3-intoxicated animals compared with control rats (Al Dera 2016).

It is of great interest that previous studies reported that there is a crosstalk between the damage elucidated by oxidative factors and inflammatory cytokines with the upregulation of matrix metalloproteinases (MMP) expression (Catania et al. 2007; Dejonckheere et al. 2011). Matrix metalloproteinases (MMPs) are from a group of Zn2 + -dependent and Ca2 + -dependent endopeptidases and unusually stated in numerous renal disorders. Prior researches have proved that MMP-9 can control extracellular matrix (ECM) deprivation throughout renal fibrosis (Kolset et al. 2012; Tsioufis et al. 2012). Therefore, MMPs have a master impact on renal tissue damage which control essential cellular actions as cell proliferation, migration, and differentiation by degrading extracellular matrix (Newby 2006).

The current study revealed that ALCL3 administration upregulated the MMP-9 m RNA expression and elevate the MMP-9-positive immunoreactivity within the cytoplasm of degenerated tubular cells. The results were confirmed statistically by a significant rise in the OD of the immune-positive appearance of MMP-9 in comparison to the control and Hesperidin groups.

Such outcomes were in agreement with the recent study, which demonstrated that the treatment of cells with ALCL3 augmented the action of MMP-9 and mRNA expression of matrix metalloproteinase-9 (MMP-9) and myosin light-chain kinase (MLCK) in a concentration-dependent way in intestinal cells. ALCL3 stimulated extracellular signal-regulated kinase 1/2 and nuclear factor-kappa B (NF-κB), resulting in mRNA expression of (MMP-9), MLCK, and inflammatory cytokines (tumor necrosis factor-alpha (TNF-α), interleukin-1β (IL-1β), and IL-6) in HT-29 cells (Jeong et al. 2020). Moreover, another work added that, in most tissues, the expression level of MMP-9 was very low but rose in response to local secretion of inflammatory cytokines and growth factors, most notably interleukin-1 (IL-1) and tumor necrosis factor-alpha (TNF-α) (Labrie and St-Pierre 2013).

Interestingly, previous studies have revealed that MMPs are implicated in initiation and progression of renal fibrosis (Zhao et al. 2013) which is produced by extreme accumulation of collagen in renal tissue that evaluated the main process responsible for the progression of chronic kidney disorders (Pradère et al. 2008).

Our findings confirmed accumulation of collagen fibers in the interstitium of renal cortex and around the blood vessels of ALCL3-treated rats which was proved by statistical analysis of area percentage of Masson’s trichrome staining of collagen fibers that revealed a significant increase compared with the control and Hesperidin groups. This finding was in agreement with other previous studies (Hanafy and Soltan 2004). Tsai et al. (2012) observed that raised MMP-9 expression in human atrophic tubular nuclear was accompanied by higher interstitial fibrosis score ($r\hspace{0.17em}=\hspace{0.17em}0.40$, $p\hspace{0.17em}=\hspace{0.17em}0.002$), signifying that it might be a protagonist in the process of renal damage. Moreover, Tan et al. (2013) demonstrated a profibrotic role of MMP-9 in tubular cell EMT. They established the pathogenesis of MMP-9’s influence to renal fibrosis via osteopontin cleavage. Correspondingly, Ling et al. (2018) reported that the atypical appearance of MMP-9 was associated with EMT in the podocytes of diabetic rats.

In contrast to our result, a prior work reported that in the model of MMP-9 deficiency mice had increased glomerular fibrin deposition and the fibrin-degrading activity of MMP-9 was clearly beneficial in this model of acute glomerular disease (Lelongt et al. 2001). Moreover, Wozniak et al. (2021) explained this phenomenon by postulating that MMP-9 activity appears to have protective roles in the acute glomerular and tubular damage phase but might be detrimental during later stages and fibrosis development.

In an attempt to further clarify the core mechanisms that might be related to the deleterious effect of ALCL3 on renal tissue was activation of apoptosis as confirmed in our study where the ALCL3-treated group exhibited a significant rise in the area percentage of FAS, caspase-3, and BAX-positive immunoexpressed cells and decrease in Bcl-2-positive immunoexpressed cells. This was in agreement with Xu et al. (2018) who stated that ALCL3 exposure stimulated Fas/Fas ligand signaling pathway, accessible as Fas, Fas ligand, and Fas-associated death domain expression augmentation and caspase-8 initiation. Furthermore, ALCL3 exposure suppressed Bcl-2 protein expression and upregulated the expressions of BAX. These results indicated that ALCL3 exposure encouraged apoptosis through activating FAS- and mitochondria-mediated signaling pathway.

In the nephrotoxic nephritis (NTN) kidneys, the ratio of Bax to Bcl-2 at protein levels was consistently elevated in the ALCL3-treated group and exhibited strong correlations with caspase-3 activity, apoptosis, inflammation, and renal fibrosis. This suggests that the Bax/Bcl-2 ratio may be significant and that it likely affects caspase-3 in mediating the apoptosis linked to inflammation and renal cell loss throughout the development of renal fibrosis. These findings were in concordance with Yang et al. (2002b)^b^.

Besides the previously mentioned mechanism of ALCL3 injury, renal tissue injury was evident upon H&E examination. Notable microscopic variations in the construction of the renal cortex were characterized by areas of tubular damage ranging from mild to severe in all treated animals, as hypertrophy and degeneration of the epithelia of the renal tubules with a distinction of the mononuclear cells’ infiltration. In this experiment, the reported histopathological changes were in agreement with the study by Sargazi et al. (2006). Similar effects have been described by Mahieu et al. (2005), who reported that aluminum had been concerned in the pathogenesis of numerous clinical problems, including renal dysfunction.

Additionally, ultrastructure examination of the renal cortex of the kidneys of animals in the ALCL3 group revealed a shorter and irregular brush border membrane, split mitochondria, and vacuolization of the cytoplasm. In the ALCL3-injected group, numerous proximal renal tubule cells exhibited thickened basement membranes, tubular epithelial damage, and changes in epithelial cell shape. This was associated with occasional tubule dilatation, interstitial tubule fibrosis, and lymphocyte infiltration into some interstitial areas.

These results were congruent with those of previously reported studies by Al Kahtani et al. (2014), who reported that ALCL3 produced ultrastructural alterations on proximal cortical tubules, the target of aluminum that exhibited nuclear-cytoplasmic changes. Necrotic nuclei and the detachment of brush borders indicated a functional impairment of urinary reabsorption.

Ultimately, the current study has proved a crucial role of Hesperidin against development of ALCL3-induced renal dysfunction. It has provided lots of evidence of the potential protective effect of Hesperidin. In fact, ALCL3 rats treated with Hesperidin displayed a remarkably improved renal function with significantly reduced serum creatinine and urea levels and uric acid in agreement with (Rushdy et al. 2012).

In an attempt to further explain the underlying molecular mechanisms of Hesperidin-induced renoprotection in ALCL3-treated rats, our study has established a vital role of Hesperidin in suppressing all the aforementioned ALCL3-induced oxidative stress, inflammatory, apoptotic, and profibrotic signaling pathways in kidney tissue which were significant and approaching the near normal healthy state of the control rats.

These findings are in accordance with those of Sahu et al. (2013), who reported that Hesperidin treatment significantly weakened the cisplatin-induced oxidative stress/lipid peroxidation and inflammation (infiltration of leukocytes and proinflammatory cytokines).

Moreover, Hanedan et al. (2018) stated that Hesperidin and chrysin could attenuate colistin-induced nephrotoxicity via antioxidant and anti-inflammatory activities. Elhelaly et al. (2019) added that Hesperidin had potent protective effects against oxidative stress, lipid peroxidation, and DNA damage induced by acrylamide-induced renal toxicity in rats.

In harmony with our results, Anandan and Subramanian (2012) reported that Hesperidin acted as a potent scavenger of free radicals in the kidney to prevent the toxic effects of gentamycin both at the biochemical and histopathological levels.

Consistent with our findings, Li et al. (2020) documented that flavonoid could impact learning and memory role by preventing extreme apoptosis and oxidative stress in ALCL3-exposed rats. Furthermore, Muhammad et al. (2019) reported that Hesperidin rescued lipopolysaccharide (LPS)-induced neuronal apoptosis by reducing the expression of associated X protein (BAX) and caspase-3 protein and promoting the Bcl-2 protein level.

In support to our results, Park et al. (2019) reported that Hesperidin improved the renal dysfunction and reduced inflammation and apoptosis after ischemia/reperfusion injury as it has potent antiapoptotic and anti-inflammatory properties due to its antioxidant property.

These results were in contrary to Aboismaiel et al. (2020) who stated the antitumor effect of Hesperidin through induction of Fas/FasL apoptotic pathway and inhibiting of Bcl-2 gene expression. Hesperidin was reported to induce apoptosis in cancer cell line by stimulating ROS-mediated apoptosis along with cell cycle arrest at G2/M phase in human gall bladder carcinoma (Pandey et al. 2019), by inhibiting Sp1 and its regulatory protein in MSTO-211H cells (Lee et al. 2012), through the mitochondrial apoptotic pathway by decreasing the expression of cyclin D1 and increasing the expression of p21 and p53 (Xia et al. 2018), and through CASP3 activation in human colon cancer cells (Park et al. 2008).

Interestingly, Hesperidin downregulated MMP-9 mRNA expression with a little positive reaction in the cytoplasm of most tubular cells and the glomerulus. The results were confirmed statistically by a significant decrease in the OD of the immune-positive appearance of MMP-9 compared with the ALCL3-treated rats.

In harmony with our results, a recent report showed that Hesperidin inhibits the expression of members of the MMP family, as reported by Lee et al. (2018), who stated that Hesperidin inhibited the MMP-9-related signaling pathway activated by UVB irradiation. Moreover, Kongtawelert et al. (2020) reported that Hesperidin significantly reduced the levels of MMP-9 and MMP-2 secreted from PD-L1 high-expressing MDA-MB231 cells. The current results were supported by these recent studies.

Remarkably, Hesperidin prohibited extreme collagen accumulation in the ALCL3 renal cortex as our results revealed that concomitant administration of Hesperidin with ALCL3 exhibited few accumulations of collagen fibers surrounding the glomeruli and around the blood vessel that exhibited a significant decrease in the area percentage of Masson’s trichrome staining of collagen fibers compared with the ALCL3 group signifying the antifibrogenic action of Hesperidin. The antifibrotic action of Hesperidin against liver fibrosis in rats has been reported in a previous study (Pérez-Vargas et al. 2014).

In further agreement, other studies confirmed that direct or indirect inhibition of MMP-9 activity by Hesperidin resulted in a decrease in renal fibrosis in obstructive nephropathy (Wang et al. 2010). Another study added that broad-spectrum metalloproteinase inhibitors have been used to treat fibrotic kidney diseases experimentally (Wozniak et al. 2021) (Abdel-Hamid 2013).

The aforementioned microscopic renal tissue degenerative changes were attenuated by the administration of Hesperidin. These results are constant with those of the study by Abd Alsalam et al. (2016), who found that the oral administration of Hesperidin (100 mg/kg and 200 mg/kg) improved the microscopic renal construction in a dose-dependent method. Moreover, Aly et al. (2017) demonstrated that treating the DEN/CCl4-induced rats with Hesperidin significantly prevented kidney injury. Additionally, Meng et al. (2020) reported that treatment with Hesperidin attenuated renal injury in I/R kidney-injured rats.

Finally, administration of Hesperidin significantly mitigated the ALCL3-induced intrarenal oxidative stress, inflammatory, and profibrotic changes and reversed all the related deleterious sequelae (Jeon et al. 2014). Our study has paid an attention to the role of MMP-9 expression as in the nephroprotective effects of Hesperidin which inhibited the MMP-9-related signaling pathway (Kongtawelert et al. 2020) activated by ALCL3 (Jeong et al. 2020).

## Conclusion

Collectively, the interpretation of our results proved tangible antioxidant, anti-inflammatory, antifibrotic, and antiapoptotic ameliorative influences of Hesperidin on the renal biochemical, cytomorphological, and immunohistochemical effects of ALCL3-induced renal damage. Furthermore, MMP-9 played a key role in the underlying molecular machinery of these results.

## Data Availability

Please contact authors for data requests (Nancy Husseiny Hassan MD—email address: nancyhusseiny@gmail.com).
